# Ethics and Fairness Considerations in AI-Based Deception Detection Technologies for Mental Health Applications: Focus Group Study

**DOI:** 10.2196/86633

**Published:** 2026-05-25

**Authors:** Sayde Leya King, Serena Bhaskar, Julia Woodward, Tempestt Neal

**Affiliations:** 1 Bellini College of Artificial Intelligence, Cybersecurity, and Computing, University of South Florida, 4202 E Fowler Ave, ENG030, Tampa, FL, 33620, United States, 1 813-396-9353; 2 Department of Mental Health Law and Policy, College of Behavioral and Community Sciences, University of South Florida, Tampa, FL, United States

**Keywords:** artificial intelligence, AI in mental health, deception detection, ethics and fairness in AI, recommendations, focus groups

## Abstract

**Background:**

Artificial intelligence (AI) technologies are increasingly being integrated into mental health settings to support tasks such as clinical documentation and decision-making. In parallel, AI-enabled deception detection, which leverages multimodal behavioral cues like facial expressions, vocal tone, and body movements, is an emerging research area. These technologies may hold relevance in mental health contexts, where deception can compromise treatment outcomes and therapeutic trust. However, most research on AI-based deception detection has focused on law enforcement domains, resulting in a limited understanding of its applicability to mental health. The ethical, relational, and practical implications of using such technologies in clinical settings remain underexplored.

**Objective:**

This study explored stakeholder perspectives on the responsible integration of AI-enabled deception detection in therapeutic contexts. We examined what ethical frameworks and safeguards are needed to guide the use of such tools in therapy (research question 1), what technical and procedural protections are necessary to uphold client confidentiality (research question 2), and what design and evaluation strategies can mitigate bias and promote fairness in clinical applications of AI-based deception detection (research question 3).

**Methods:**

We conducted 6 virtual focus groups (n=18) with individuals who were both mental health clinicians and current therapy clients. Participants responded to a hypothetical scenario describing the integration of AI-based deception detection into therapy. A semistructured guide was used, and transcripts were analyzed thematically using a combination of inductive and deductive coding strategies.

**Results:**

Participants expressed a range of concerns about the integration of AI-enabled deception detection in therapy, highlighting potential ethical, relational, and contextual challenges. In response to research question 1, participants described fears of a “Big Brother” atmosphere and distractions from in-session notifications. However, many viewed telehealth as a less intrusive context and emphasized respecting disclosure timing and maintaining client agency. For research question 2, participants raised concerns about unconscious data capture, subpoena risks, and unclear data protections. For research question 3, participants cautioned that such tools may exacerbate power imbalances, erode trust through false positives, and lack cultural or contextual sensitivity. Informed by these findings, the research team developed design and policy recommendations, including minimizing in-session notifications; ensuring ongoing consent; establishing transparent data policies; training models on diverse populations; exploring modeling personalization; and developing equitable use policies.

**Conclusions:**

While AI-enabled deception detection technology holds promise for augmenting clinical insight, its integration into therapy must be guided by a commitment to safe, ethical practice. Researchers and clinicians should collaborate to design systems that (1) integrate seamlessly into therapy without disrupting therapeutic relationships, (2) prioritize data security and transparency to protect client confidentiality, and (3) implement fairness safeguards that address cultural representation and power dynamics. Addressing these challenges is essential to ensure that AI-based deception detection enhances, not undermines, therapeutic practice.

## Introduction

Deception, or the intentional withholding or misrepresentation of information [1,2], challenges core practices across a range of fields, including law, government, workplace environments, and health care. In occupational settings, deception may result in outcomes like job loss or rescinded offers [3], while in public domains such as media or government, the consequences of deception can be more variable and context dependent [4]. In health care, particularly in mental health, deception poses unique challenges due to the deeply relational nature of clinical work. Mental health treatment relies heavily on trust, psychological safety, and open disclosure between clients and clinicians. However, previous studies indicate that even trained professionals often demonstrate limited and variable accuracy when detecting deception, relying primarily on judgment and observable cues such as body language, verbal inconsistencies, and visual incongruence [5,6]. This limitation raises serious concerns—when clients withhold or distort information, it can undermine therapeutic trust and compromise treatment effectiveness [7,8]. In extreme cases, undetected deception can have fatal consequences—for example, many individuals who have died by suicide had not disclosed suicidal ideation during clinical encounters [9,10]. These realities highlight the urgent need for more reliable, evidence-based tools to support deception detection in mental health care.

Automated deception detection has emerged as a promising approach for identifying potentially misleading or withheld information. These systems use artificial intelligence (AI) to classify patterns in behavioral cues, such as eye gaze, facial expressions, body gestures, vocal tone, speech patterns, and physiological signals in deception-related classification tasks [11]. The diagnostic value of individual cues remains contested in the deception literature, underscoring the importance of cautious interpretation and responsible deployment.

To date, such systems have been primarily developed and tested in high-stakes contexts where the consequences of deception can be significant or irreversible, such as in legal investigations, forensic interviews [11], travel and border security screenings [12], and employment evaluations [13]. In these domains, deception detection tools aim to support decision-making where human judgment may be insufficient or inconsistent. Their use in therapeutic contexts, however, remains largely unexplored. Since therapy involves highly sensitive disclosures, this is a critical gap; undetected deception, whether intentional or unintentional, can undermine diagnosis, treatment planning, and client safety. Moreover, therapeutic settings introduce unique relational and ethical dynamics that distinguish them from other high-stakes environments. These differences raise important questions about whether, and how, AI-enabled deception detection could be ethically and effectively adapted to support mental health care. While such tools may offer opportunities to augment clinical insight, their integration into therapy also presents serious ethical, relational, and fairness challenges, particularly given the deeply personal and trust-based nature of mental health treatment.

There is a growing interest in the use of AI applications for mental health care [14], primarily focusing on supportive or administrative tools, such as note-taking assistants or conversational agents. However, such applications are yet to perform behavioral inference in real time. This distinction is critical; AI tools that merely assist or reflect pose different relational risks than those that actively interpret client behavior, such as in detecting deception. For example, Haber et al [15] examined client interaction with generative AI chatbots designed to support self-reflection through imagery and conversation. While some participants reported that these “artificial third” agents reinforced the therapeutic alliance, others experienced moments of empathic failure. In response, the authors proposed the Screening, Alignment, Facilitation, and Evaluation of AI-Enhanced Interventions (SAFE-AI) Protocol, a set of guidelines emphasizing transparency, patient safety, and stakeholder engagement for therapeutic AI design. However, such frameworks primarily address AI as a reflective companion, not as an active evaluator of client honesty or intent. This leaves a gap in guidance for more interpretive applications, like AI-enabled deception detection, where the stakes for client autonomy and trust are significantly higher. Furthermore, while deception in therapy has been studied for its clinical relevance, such as understanding when and why clients withhold information [6,8,16,17], there has been little exploration of how AI might reliably, ethically, and complement this work.

In our previous work, we began exploring this space in a qualitative interview study consisting of 15 mental health professionals, examining their experiences with client deception and their perspectives on the use of AI for deception detection in therapeutic contexts [5]. Participants in that study expressed serious concerns about the potential erosion of client autonomy and trust and anticipated negative client reactions to the use of such technologies. However, that captured only clinician perspectives, leaving client views inferred rather than directly represented.

To extend this line of inquiry and more fully inform the ethical design of AI-enabled deception detection tools by incorporating both clinician and client perspectives, this study involved 6 focus groups with individuals who uniquely occupy dual roles as both practicing clinicians and active recipients of therapy. This design enabled direct access to first-person client experiences while preserving the clinical context necessary to interpret how such technologies might intersect with therapeutic practice. Rather than positioning participants as proxies for all clients or clinicians, their dual roles provided reflexive insight into how professional assumptions align—or diverge—from lived therapeutic experience. This study is guided by three research questions:

1. What ethical frameworks and safeguards are needed for the responsible integration of AI-enabled deception detection in therapeutic settings?
2. How can client confidentiality and trust be preserved when deploying AI tools in therapeutic contexts?
3. What design and evaluation strategies can prevent bias and promote fairness in therapeutic applications of AI-based deception detection?

By exploring these issues through qualitative inquiry, this study contributes to a growing body of research [18-21] on ethical AI in mental health and offers early guidance for developers, clinicians, and policymakers seeking to responsibly navigate the use of high-stakes behavioral inference tools in therapeutic settings.

## Methods

### Recruitment

We recruited 18 adult mental health clinicians who had provided therapy within the past 5 years and were also actively receiving care for their own mental health or well-being. Participants were recruited based on their dual roles to enable reflexive comparison between professional assumptions about therapeutic practice and first-person lived experience as a therapy client. Eligibility was assessed via an online screening survey; eligible individuals were directed to an availability form to facilitate focus group scheduling. After each focus group, participants completed a 20-item questionnaire collecting demographic information, professional counseling experience, client population demographics, and comfort levels with technology. Demographic summaries are presented in Table 1, with individual participant details provided in Multimedia Appendix 1.

**Table 1.** Participant characteristics (n=18).

Characteristic	Participants, n (%)
Race or ethnicity	
Black or African American	5 (27.7)
White or Caucasian	8 (44.4)
Hispanic or Latino	3 (16.7)
Biracial	2 (11.1)
Total	18 (100)
Age group (y)	
18‐25	3 (16.7)
26‐35	11 (61.1)
36‐45	3 (16.7)
46‐55	1 (5.5)
Total	18 (100)
Gender	
Women	16 (88.8)
Men	2 (11.1)
Total	18 (100)
Mental health challenges^a^	
Yes	16 (88.8)
Anxiety	5 (27.7)
Depression	6 (33.3)
Trauma	3 (16.7)
Attention-Deficit/Hyperactivity Disorder	5 (27.7)
Undisclosed	2 (11.1)
Total	18 (100)
Student status	
Yes	7 (38.9)
No	11 (61.1)
Total	18 (100)

a Categories of mental health challenges are not mutually exclusive; participants could report multiple conditions. Seven participants reported more than one mental health challenge.

### Focus Groups

We conducted six 90-minute virtual focus groups between September 12, 2024, and March 6, 2025, using Microsoft Teams, which were all audio- and video-recorded and transcribed using Teams’ built-in transcription feature. To support open participation, particularly in smaller and dyadic sessions, facilitators established clear ground rules at the outset (provided in the focus group guide in Multimedia Appendix 2), used open-ended prompts, and used structured turn-taking to ensure all participants had equal opportunity to share, consistent with established guidelines for conducting focus groups [22]. Each focus group followed a semistructured protocol consisting of a hypothetical scenario, 9 key questions, and a series of follow-up prompts designed to explore participants’ reactions to the ethical implications of AI-enabled deception detection. The scenario described an AI system capable of identifying potential deception during therapy by analyzing multimodal behavioral signals. Participants were encouraged to consider and reference this scenario in their responses. In our first focus group, we noticed participants provided several details regarding how the technology might be implemented in real time. To refocus participants on the ethical and safety considerations of the technology, we provided examples, such as a Google Home or smartwatch, to anchor the conversation. The focus group guide with the updated text highlighted in yellow is provided in Multimedia Appendix 2. The focus group guide began with questions prompting participants to reflect on their personal ethical values and how those values align, or diverge, from those they uphold in their professional roles. It then transitioned into targeted discussions about the ethical, relational, and practical considerations of integrating AI-based deception detection into therapy, with particular attention to issues such as client autonomy, data access, and fairness.

Focus group sizes varied; the first session included 5 participants, the second had 3, sessions 3-5 were dyadic, consisting of 2 participants each, and the final session had 4 participants. This variation was not an intentional design decision but arose from recruitment and engagement challenges common in qualitative research with specialized professional populations. Similar difficulties have been documented in recent focus group–based research, which has attributed low participation to factors such as virtual meeting fatigue, competing time demands, limited availability of participants in specialized populations, and the sensitive nature of the topics discussed [23,24]. Despite this variation, data collection continued until thematic saturation was reached, indicating that additional sessions were unlikely to yield substantively new insights.

### Qualitative Analysis

Two independent raters, authors SLK and SB, conducted deductive coding on the first 3 focus group transcripts, guided by the study’s research questions and the codebook developed from our previous study [5] involving interviews with 15 mental health professionals on their general perceptions regarding using AI-based deception detection technology in therapy. These initial transcripts included both dyadic and larger group sessions. After this initial round, the raters met to discuss discrepancies and collaboratively refine the preliminary coding structure. This revised structure was then applied to the remaining transcripts through an iterative process of independent coding, with the codebook refined as needed. No substantive differences in theme generation were observed between dyadic and larger group sessions. Coding continued until thematic saturation was reached, which was achieved with the sixth focus group transcript.

Throughout the coding process, we systematically assessed the alignment between the emerging codes and our research questions to ensure that the focus group guide elicited rich, relevant data. This ongoing evaluation confirmed the guide’s adequacy in capturing content central to the study’s objectives. Once the coding structure was finalized, all previously coded excerpts were reviewed and organized into major-minor theme pairs. A final round of independent coding was conducted to ensure consistency. To assess interrater reliability across all transcripts, we calculated Cohen κ, a statistical measure of agreement between coders beyond chance, which yielded a value of Cohen κ=0.909, indicating strong agreement [25]. A copy of the final coding structure with definitions is available in Multimedia Appendix 3. Additionally, we provide a consolidated frequency table including corresponding participant IDs and the number of students and nonstudents who mentioned each code in Multimedia Appendix 4.

### Ethical Considerations

Recruitment materials (eg, email invitations and flyers) were distributed through online platforms (eg, TherapyDen [26] and PsychologyToday [27]), US university counseling centers, academic listservs, and direct outreach to faculty and staff in psychology, social work, and counseling via email, phone, and LinkedIn. Interested individuals were directed to an online consent form that fully described the study’s purpose and participation requirements. Informed consent was required before continuing. Because participants were invited to reflect on their lived experiences as both clinicians and therapy clients, the research team gave careful consideration to the potential sensitivity of these discussions. The consent form and focus group guide emphasized that participation was voluntary, that individuals could decline to answer any question or withdraw at any time without penalty, and that identifying information would be removed from transcripts before analysis. During the focus groups, facilitators clarified that participants were not expected to disclose personal clinical details but could respond from either a professional or general client perspective. Participants were also reminded that confidentiality in a focus group cannot be absolutely guaranteed and were asked to respect one another’s privacy. These safeguards were intended to minimize emotional risk while preserving participant autonomy and ensuring that discussion remained centered on ethical reflection rather than personal therapeutic disclosure. Participants received a US $25 electronic gift card upon completing a focus group and demographic questionnaire. This study was approved by the University of South Florida’s Institutional Review Board (STUDY007541).

## Results

### Sample Characteristics

All participants completed a brief demographic questionnaire. In total, 44% (8/18) of participants in this study identified as White or Caucasian, 27% (5/18) as Black or African American, 16% (3/18) as Hispanic or Latino, 11.1% (2/18) as Biracial. The sample was majority women-identifying participants (n=16) with 2 men-identifying participants. Most participants (12/18, 61%) were between the ages of 26 and 35 years with an average age of 31.9 (SD 7.27) years. Furthermore, 2 participants chose not to disclose if they were experiencing mental health challenges in the demographic questionnaire. Of the 16 participants who did disclose, 7 (38%) identified as having more than 1 mental health challenge. Commonly disclosed mental health challenges included depression (n=6 participants), anxiety (n=5 participants), Attention-Deficit Hyperactivity Disorder (n=5 participants), and trauma (n=3 participants). We attribute the gender imbalance of our sample to the gender imbalance inherent to the mental health profession per the American Psychological Association [28].

Participants also completed a questionnaire aimed at gauging comfort levels with different technologies. Average comfort levels are summarized in Table 2. On average, participants were least comfortable with generative AI chatbots (mean 2.8, SD 1.05) and most comfortable (mean 4.5, SD 1.14) with online browsing, videoconferencing, and online communication (mean 4.5, SD 0.78). Although there was some variability in responses, the higher mean comfort rating for internet browsing indicates that most participants were generally comfortable with this technology. This suggests that familiarity with routine digital tools was high across the sample, even as individual comfort levels varied.

**Table 2.** Participant comfort levels with different modes of technology.

Technology	Mean (SD)
Videoconference and online communication	4.5 (0.78)
Electronic health records systems	4.3 (0.84)
Computers and operating systems	4.2 (0.81)
Internet browsing	4.5 (1.14)
Productivity software	3.7 (1.01)
Generative AI^a^ chatbots	2.8 (1.05)

a AI: artificial intelligence.

The higher SD for generative AI chatbots suggests that some participants are generally comfortable with this technology, despite a low average comfort level rating.

### Qualitative Findings

#### Thematic Overview

Our analysis of participants’ perspectives revealed 3 major themes, including *integration*, *data confidentiality*, and *AI integrity*, each reflecting core concerns about the ethical and practical implications of AI-based deception detection in therapy. These themes were supported by 13 minor themes and a total of 53 unique codes. Together, they reflect the diverse ways participants articulated potential risks, necessary safeguards, and design priorities. In the sections that follow, we present each major theme in turn, drawing on participant quotes to highlight key insights and their implications for future development and use of such technologies in clinical settings.

#### Integration

##### Overview

The major theme of integration encompasses the direct aspects of incorporating AI-based deception detection technology into therapeutic practice. It includes 7 minor themes*—disruption*, *client autonomy*, *power imbalance*, *use considerations*, *practice considerations*, *therapeutic environment*, and *stigma*. The frequency of codes in this major theme is provided in Table 3.

**Table 3.** Minor themes, codes, and frequency by participant for the major theme integration.

Theme and code	Participants, n (%)
Disruption	
Therapeutic	12 (66.7)
Physical	3 (16.7)
Client autonomy	
Self-paced disclosure	14 (77.8)
Client choice	8 (44.4)
Right to lie	7 (38.9)
Client disclosure	7 (38.9)
Client awareness	5 (27.7)
Client only use	1 (5.5)
Power imbalance	
Client-clinician	11 (61.1)
Client-AI^a^	5 (27.7)
Client-parent	2 (11.1)
Clinician-AI	1 (5.5)
Use considerations	
Preexisting conditions	9 (50)
Setting	8 (44.4)
Real-time decision making	5 (27.7)
Postsession workflow	5 (27.7)
Assessment assistant	3 (16.7)
Topic selection	2 (11.1)
Client selection	2 (11.1)
Practice considerations	
Clinical responsibility	9 (50)
Merit	9 (50)
Clinician bias	6 (33.3)
Clinician qualification	5 (27.7)
Equal availability	3 (16.7)
Therapeutic environment	
Telehealth	5 (27.7)
Big brother	4 (22.2)
In-person	3 (16.7)
Stigma	
Help-seeking	4 (22.2)
Clinician self-stigma	1 (5.5)

a AI: artificial intelligence.

##### Disruption

The minor theme of *disruption* captures participant concerns about the potential for AI-based deception detection technologies to interfere with the therapeutic process. This theme emerged across all focus groups. Moreover, 2 participants, speaking from the clinician’s perspective, emphasized that receiving deception alerts during a session could disrupt the flow of interaction, making it difficult to remain fully present or engaged with clients.

*Kind of having to check said device for that notification…could potentially throw off the flow…might be flowing really well…[and] you get that notification and it could throw you off.*

[P16, FG 5]

Furthermore, 8 participants also shared concerns that the therapeutic alliance could be negatively impacted by deteriorating client trust.

*Being a counselor who really prioritizes the therapeutic relationship and the trust that’s built between the client, that would be my biggest concern and just how this would impact their ability to trust and feel safe with me.*

[P9, FG 2]

Speaking from their experiences as clients, 12 participants expressed that this integration could be uncomfortable and create an additional barrier to them engaging with their clinicians.

*As a client, actually, I won’t really feel comfortable…sometimes I actually don’t want to share things…[or on] second thought I should share this with you in a different manner.*

[P3, FG1]

*I think that can definitely harm the relationship. So yeah, I don’t think I’d be quite as trusting of my therapist if that were the case…I’d be less inclined to be authentic.*

[P9, FG 2]

##### Client Autonomy

The minor theme of *client autonomy* reflects participants’ emphasis on respecting the client’s right to self-determination. This theme appeared in each focus group and was closely tied to clinicians’ theoretical orientations, many of which prioritize the belief that clients are the experts of their own experiences. Participants expressed that clients should have the right to decide if and when the technology would be used during sessions and should be fully aware of its presence and purpose. From the clinician’s perspective, several participants (n=7) highlighted the importance of clear, thorough disclosure through an active and ongoing informed consent process. Moreover, 8 participants further emphasized that consent should not be treated as a one-time event, but rather as something that may evolve based on the session’s content or the client’s comfort level.

*If a client was like, yeah, I want to use this…it would have to be a client-directed decision.*

[P17, FG 5]

*You would have to ensure that a client’s autonomy doesn’t feel violated with this from a standpoint of being able to consent, understand what’s happening with their data and understand what, why and how the data is being collected. And creating like a very open space around like this is what this is used for.*

[P13, FG 3]

Nearly all participants (n=14) also emphasized that clients have the right to withhold or distort information as part of their therapeutic journey. They noted that the goal of therapy is not to uncover absolute truths but to empower clients by honoring their autonomy and recognizing them as the experts of their own experiences. Gradual disclosure, sharing more as trust builds, is a fundamental aspect of the therapeutic process that allows clients to “own their truth” in their own time. Some participants (n=4) also highlighted that deception could serve as a protective mechanism for clients, especially when discussing difficult or traumatic topics. Notably, participants reflected on these dynamics both as clinicians and from their own experiences as clients.

*I know that I have lied in therapy…it’s usually for some form of protection.*

[P1, FG 2]

*It’s not our job to be the truth police.*

[P18, FG 6]

*So much of our training is people are the expert in their own life and in their own existence. And I think about, you know, something that you said too about how there’s so many other reasons why people would not necessarily be truthful, right?*

[P15, FG 4]

##### Power Imbalance

Therapy is often structured around the perception of clinicians as experts, which can create an inherent *power imbalance* between clinicians and clients. This theme was present in all focus groups, capturing participant reflections on this dynamic and how the integration of AI-enabled deception detection technology could potentially influence, or exacerbate, existing asymmetries in the therapeutic relationship. Several participants (n=11) also discussed broader power dynamics that can emerge throughout the therapeutic process, particularly in relation to authority, control, and trust.

*I fear it would put the clinician in a little bit of like a expert role and at least like in my social work training, like we try really hard not to be expert on people’s lives and to let them be the expert on their lives.*

[P14, FG 4]

*I still think I would go into my sessions not feeling as safe because I do feel like my therapist would have some sort of…power over me.*

[P10, FG 5]

*Just keep thinking of this tool as…reinforcing a power differential. The implications…are obviously worse [for] more vulnerable populations.*

[P17, FG 5]

A couple of participants (n=2) also shared concerns about the impact of the technology on vulnerable populations like minors.

*It still [creates] a kind of like pressure almost for them to feel the need to come clean with that information before they’re ready to come forward with that information. And it just also makes me wonder about, like, the how it could impact minors. Like if parents were to want that for their child, but like the child might not want that tool used. So I could see like some conflict with that.*

[P10, FG 6]

Interestingly, participants often described the technology as a “third party” in the therapy room. They noted that this presence could lead to frustrations, not just with the clinician or the process, but with the technology itself. Some participants (n=5) expressed concern that the technology might inadvertently overshadow the client’s voice, thereby disempowering them and shifting the therapeutic focus away from the client’s narrative.

*What if it alerts that I’m lying about something and I’m not?*

[P8, FG 2]

*If this thing is beeping at me…I’m being honest here, so that would also like introduce a layer of almost like a third entity in the room. It’s like I’m not mad at myself. I’m not mad at my therapist. I’m like mad at this AI thing.*

[P14, FG 4]

An interesting perspective emerged around the potential for the technology and the clinician to appear in conflict, which a participant suggested could undermine client confidence in the technology and negatively affect their perception of the clinician.

*Can you imagine if the AI gets it wrong and then now you have like a splitting problem? Well, AI said X…like how unprofessional would that be?*

[P5, FG 6]

##### Use Considerations

Participants shared a range of insights about when and how to use deception detection technology, including decisions about which clients to use it with and how to interpret and act on its findings. This minor theme, *use considerations*, was present in all focus groups. Some participants (n=5) noted they would engage with the information in real time, responding to indications of potential deception during the session, while others (n=5) suggested it might be more appropriate to reflect on such insights outside the immediate therapeutic context, depending on timing and clinical judgment.

*It maybe a little bit more practical or appropriate if you’re just being open with your client and saying like, “Hey…I’m getting an indication that there might be some deception here. Let’s walk through that.” In that case [the client] can kinda correct you, like “your question really shocked me and I was wasn’t sure how to answer.” Oh, OK.*

[P2, FG 1]

*A pretty experienced clinician might have the level of clinical discernment and skill to choose when to not bring that into session and…[decide] what to do with that information and how that informs treatment.*

[P14, FG 4]

A few participants (n=3) also discussed alternative use cases for the technology’s insights, such as using it as a clinical aid during assessments of disorders or symptoms. They highlighted its potential value in specific settings or with clients experiencing particular conditions, where information about possible deception could support diagnostic or treatment decisions.

*For example, when I’m doing a risk assessment to determine if someone [requires] involuntary hospitalization. Like of course I want accurate information. If someone’s telling me “No, I’m not stockpiling medications” and they are, it’d be great if I could just know that they’re being dishonest with me in those circumstances.*

[P14, FG 4]

*For assessments around their trauma symptoms related to the assault…I feel like a tool like this could be a super helpful, especially in sessions where we are scoring and going over the assessments…Like how accurate or how truthful this kiddo is being within answering these questions?*

[P13, FG 3]

One participant noted the opportunity to leverage deceptive insights to track their own accuracy at detecting client deception and their response to this deception.

*Less using the data [as] a gotcha for the client and more of “how am I responding to deception?” I knew they were lying in the moment too. Like it caught it there. I knew it and I felt uncomfortable or being able to just listen back and look back and see how you were responding as a counselor.*

[P17, FG 5]

Finally, participants explored the possibility of selectively using the technology, such as enabling it only during discussions of certain topics or with specific clients. A couple of participants (n=2) raised ethical concerns about fairness, questioning whether it is appropriate to use deception detection with some clients but not others. Others (n=3) emphasized that the technology may not be suitable for all clients or therapeutic contexts, highlighting the need for careful, individualized consideration.

*Does that come down to picking and choosing which clients it’s actually best to use it with? Because I can think of several that I’ve worked with in the past that this would not have been good…Coming back to the fair point of is it fair that me as the clinician decides?*

[P1, FG 2]

##### Practice Considerations

The minor theme of *practice considerations* encompasses reflections on clinicians’ professional responsibilities, clinical judgment, and the broader distribution of the technology. This theme was present in all focus groups. A few participants (n=5) discussed how integrating deception detection could influence clinical decision-making and raised questions about the level of experience or training necessary to interpret and apply the technology’s insights effectively and ethically.

*Green clinicians…might not know what to do with this level of information.*

[P14, FG 4]

*This would affect both parties…I’d be in my head about this.*

[P9, FG 2]

*This could make you a little lazy as a therapist.*

[P5, FG 6]

Highlighting the responsibility clinicians bear regarding the safety of their clients, half of the participants in this study (n=9) considered how integrating the technology could impact that responsibility.

*It would be greatly great for me to know…There’s been the number of calls where I have had to sit with that ambiguity…I feel nervous, but I have to protect and say like, “OK, I have to put in your hands,” which is a hard space for crisis counselors to be in.*

[P6, FG 3]

*But in the same way that if safety comes up, that kind of takes precedence. And what happens to you after this session?*

[P13, FG 3]

Because clinical responsibility includes mitigating harm, participants emphasized the need to evaluate the integration’s potential beneficence. However, others (n=9) remained unconvinced that its benefits would outweigh the potential for harm.

*But for me it’s beneficence of as a clinician, my first thought is how can I do the most good for them and keep them from the most harm when it comes to being in my sessions?*

[P1, FG 2]

*Is it really in the best interest of the client? Most often, the answer is no.*

[P17, FG 5]

##### Therapeutic Environment

The minor theme of *therapeutic environment* reflects participant concerns about how AI integration might affect the overall atmosphere of therapy. This theme was discussed in 5 of the 6 focus groups. Participants frequently questioned the feasibility of implementing such technology across different therapeutic contexts and expressed discomfort with the sense of being monitored. Some participants (n=5) noted that AI-enabled deception detection may be more appropriate in telehealth settings, while its use in in-person sessions could feel intrusive or unsettling.

*I could see it more in like a telehealth…But I have a really hard time seeing it [in-person].*

[P1, FG 2]

*I think over telehealth it could be fairly seamless.*

[P12, FG 1]

From the client perspective, 4 participants described the technology as a “big brother,” “another presence,” or “third person in the room,” referring to the feeling of being observed or monitored in a “pervasive” or invasive manner.

*It would be like a watchful eye in the room almost that there’s some kind of, like [need to] right answer in a way. I feel like it just sort of ups the stakes…*

[P14, FG 4]

##### Stigma

Although not raised in every group, participants in 3 of the 6 focus groups expressed concerns that integrating deception detection technology could increase *stigma*, discourage help-seeking, or create new barriers to accessing mental health care.

*It might actually reduce the rates of clients coming to see a therapist.*

[P3, FG 1]

*[It] could add another layer to delay people from seeking help.*

[P4, FG 1]

Furthermore, 5 participants reflected on both self-stigma and external stigma related to mental health challenges, help-seeking behaviors, and even the mental health profession itself.

*Even if you do a bad job…you hope you don’t leave your client with a feeling of, “I’ll never try therapy again.”*

[P14, FG 4]

*It bring[s] stigma and misunderstanding…someone can say, “your job is easily replaceable by AI.”*

[P8, FG 2]

### Data Confidentiality

#### Overview

The second major theme, data confidentiality, captures participants’ concerns about the sensitive information that AI-enabled detection technology could generate. A key issue raised was the uncertainty around whether such data could be reliably kept confidential. Participants emphasized the importance of strict compliance with federal regulations governing confidentiality, as well as careful attention to all aspects of data handling, including sharing, storage, and usage. Minor themes emerging from this major theme include *data access*, *data storage*, *regulatory compliance*, and *data use*. Corresponding code frequencies are provided in Table 4.

**Table 4.** Minor themes, codes, and frequency by participant for the major theme of data confidentiality.

Theme and code	Participants, n (%)
Data access	
Information sharing	10 (55.5)
Security	8 (44.4)
Client data security	5 (27.7)
Clinician data security	2 (11.1)
Information ownership	1 (5.5)
Data storage	
Data translation	8 (44.4)
Information retention	7 (38.9)
EMRs^a^	6 (33.3)
No storage	4 (22.2)
Private practice EMR	1 (5.5)
Regulatory compliance	
HIPAA^b^	6 (33.3)
Ultimate regulation	3 (16.7)
Cures Act	1 (5.5)
General regulation	1 (5.5)
Data use	
Legal contexts	8 (44.4)
Market contexts	2 (11.1)

a EMR: electronic medical record.

b HIPAA: Health Insurance Portability and Accountability Act.

#### Data Access

*Data access* was important to participants in many ways to protect the client and their right to confidentiality and privacy. This minor theme was present in all 6 focus groups. Participants (n=2) suggested that access to the deception detection technology’s data could be limited to the clinician alone, while others (n=2) suggested that data access could go beyond the clinician but remain limited.

*I’d want to limit [access] as much as feasibly possible…really just the clinician.*

[P6, FG 3]

*Maybe access to the results looks like clinician and maybe one other person…not like everybody and their mother.*

[P13, FG 3]

The topic of data security was popular, with participants (n=5) emphasizing the importance of cybersecurity, in particular. This typically involved concerns of unauthorized access to the session through some vulnerability in the deception detection technology.

*I assume it would be looking at like a body language and tone and things like that? So there would be some element of recording. What would happen if someone hacked into a therapy session? As a client…That’s like a horrible, horrible fear.*

[P18, FG 6]

*I feel like working in such a big university setting, they’re so serious about certain software that we’re using…I would definitely, you know, be a little concerned about can this data somehow be accessed. Is it truly confidential?*

[P9, FG 2]

One participant compared this security and data access challenge to what the field has experienced regarding scribe technology.

*We already have difficulty keeping confidentiality with the technology that we have in use between electronic medical records. Different devices and options for scribe technology to be able to write your notes for you. Listening to that right, and the potential for it to be hacked, et cetera.*

[P16, FG 5]

Participants (n=5) were also adamant about protecting the client’s data. Of these 5 participants, 2 elaborated and shared the importance of protecting themselves as clinicians as well.

*There always may be a opportunity for the data to be retrieved in some [fashion] that you may not be able to completely protect yourself or the client.*

[P4, FG 1]

*One of those good points of like insurance not only for the client but also for the therapist.*

[P13, FG 3]

#### Data Storage

Participants shared their views on how the output from deception detection technology should be stored—if at all. *Data storage* was discussed in all focus groups. Throughout the focus group sessions, participants discussed what information, if any, should be retained, how long it should be stored, and whether it should be integrated into electronic medical records (EMRs) like other behavioral health data. Some participants (n=4) argued against storing any client data from the technology to better protect client privacy.

*I’d prefer it be kept nowhere.*

[P14, FG 4]

*No data storage would be something that I feel like I would be more comfortable with…right, it was deleted — but I can also grab my phone and look at the deleted voicemails I have.*

[P18, FG 6]

Several participants (n=8) also voiced concerns surrounding how much information is available in the stored data, primarily citing concerns of the data being leveraged in a way that could harm clients.

*I do work with my clients’ lawyers sometimes and I have to be really conscientious of the way that I do document things. Especially when you [are] talking about the ethics of other agencies.*

[P18, FG 6]

Participants had mixed opinions about storing data indicative of deception in a client’s EMR (eg, Epic and myChart). One participant expressed concerns that incorporating implicit behavioral data into a client’s record could be unethical, as it might include unintended or unverified information. Others (n=8) suggested that any stored outcomes should be narrowly defined and limited to tangible findings rather than raw data.

*Most of the things that are coming to mind are ways in which having that around as part of the record feels a little scary and potentially unethical.*

[P14, FG 4]

*Yeah, I’m thinking if this was stored in some way in an EMR I can see that being helpful…Usually you want to translate raw data…into something that’s more relevant or understandable to the treatment plan. But that file itself, I don’t know if that would really be needed if that would need to be stored in anyway.*

[P9, FG 2]

If the data were to be stored, some participants (n=7) raised questions about how long it would remain accessible in an EMR system and shared concerns about the implications of long-term availability.

*I know, like in my master’s program, when we had to record our sessions of clients…there was a cloud [to store] the recordings which is automatically deleted after 30 days. So maybe something like that? But again, I’d still have those concerns of does it actually get deleted?*

[P9, FG 2]

*If a client knows that this is not just going to hang out in a cloud somewhere forever and ever, that it’ll be deleted after like a week or something…informed consent is like. This is why we do this. And then this is how we use that data.*

[P5, FG 6]

#### Regulatory Compliance

As behavioral health professionals, clinicians are bound by several federal regulations pertinent to confidentiality. This minor theme, *regulatory compliance*, was present in 5 focus groups. Participants discussed their thoughts on the regulation of the data generated by deception detection integration. Some (n=6) argued it should be regulated in a similar fashion as their current session notes or other health records by the Health Insurance Portability and Accountability Act (HIPAA) [29] and the Cures Act [30].

*I think again with confidentiality, you would just have to feel very assured that not only is [it] HIPAA compliant…there’s the Cures Act where you now have to release like medical notes or information like right away. It would have to be compliant with like national and then state laws in terms of how clients are receiving their own information.*

[P12, FG 1]

*As long as the information was hosted on a HIPAA compliant server, you know, like a hosting platform, then I could see that being okay.*

[P5, FG 6]

Others (n=3) argued that given the implicit nature of the signals potentially used as input, the data should be regulated extensively, surpassing current regulatory standards for typical health care data.

*This feels like even more precious in some ways, because it’s not even like a conscious report. It’s an unconscious piece of data. And so I imagine that maybe it would even need a higher level of protection.*

[P14, FG 4]

#### Data Use

The minor theme of *data use* was present in 4 focus groups. Although this study examines the integration of automated deception detection technology in mental health, clinicians interact with different systems where their clinical evaluation of client treatment is relevant, such as the court for mandated clients or clients dealing with custody battles.

*God forbid something gets subpoenaed into court. That’s not even like a conscious utterance that you made. I think that there’s some like concerns about the potential implications of that from a legal standpoint.*

[P14, FG 4]

*If there’s like larger potential ramifications, right? Like in court, Who’s getting custody, in children services? [In] prisons…Yeah, that feels way riskier.*

[P12, FG 1]

Finally, in the era of big data, the following participants cautioned against the mining of the data for financial gain:

*Like so many things with AI, it’s like so much data mining…Who all is the end user of the data that it gathers?*

[P18, FG 6]

*Depending on whoever is hosting this platform…if they can find a way to make money off of this information…*

[P5, FG 6]

### AI Integrity

#### Overview

The third major theme, AI integrity, includes general concerns from participants about the use of AI-enabled deception detection. Many of these concerns stemmed from limited confidence in such technological ability to perform deception detection in a reliable and robust manner. Participants also expressed their own personal fears regarding AI and technology altogether. Minor themes emerging from this major theme include *robustness of AI* and *trustworthy AI*. Corresponding code frequencies are provided in Table 5.

**Table 5.** Minor themes, codes, and frequency by participant for the major theme AI^a^ integrity.

Theme and code	Participants, n (%)
Robustness of AI	
Detection cultural bias	13 (72.2)
Detection fidelity	11 (61.1)
Generalized model	7 (38.9)
Personalized model	7 (38.9)
Model improvement	4 (22.2)
Contextual use	2 (11.1)
Trustworthy AI	
Mistrust of technology	3 (16.7)
AI anxiety	2 (11.1)

a AI: artificial intelligence.

#### Robustness of AI

Confidence in predictions and scoring is critical in any AI application, and this need is amplified in therapeutic contexts. This minor theme, *robustness of AI*, was discussed in all focus groups. Participants noted challenges such as the physical manifestations of psychological disorders and the inherent discomfort of certain therapeutic modalities. They cautioned that these signals could easily be misinterpreted by an AI system as indicators of deception.

*Is it picking up on deceit or dishonesty—or just discomfort?…like [eye movement desensitization and reprocessing (EMDR)] where you’re purposefully uncomfortable…is that going to be misinterpreted as deceit?*

[P12, FG 1]

*Therapy involves kind of stepping out of your comfort zone…we talk about difficult things. That just might really elicit physiological responses.*

[P2, FG 1]

Cultural considerations and ensuring robustness across diverse groups were primary concerns for participants (n=13), particularly regarding challenges related to the representativeness of the training sample.

*What samples was this normed on? Was it one demographic or across cultures? To be fair, we need a good representation.*

[P9, FG 2]

*AI data is…a large majority [is] coming from white men. That’s a huge problem.*

[P17, FG 5]

Several participants (n=11) argued that limited representation during training of an AI would, in turn, limit its ability to understand the nuances, subtleties, sarcasm, and exaggerated expressions of clients, which are unique to individuals, cultural groups, and more.

*The AI is not…smart enough…to make those discernments. Body language matters. Tone inflection matters. Eye contact matters.*

[P16, FG 5]

*I think about people that have a lot of sarcasm…it may be perceived they’re being dishonest when they’re just being sarcastic.*

[P15, FG 4]

In total, 7 participants suggested that a model could potentially learn about the client over time, nodding to personalized deception detection models.

*People are different and they change. And so I just really struggle with thinking about like as a client. What would be fair?…How would it be able to keep up with my growth and ensure that kind of fairness? I don’t know.*

[P15, FG 4]

#### Trustworthy AI

The minor theme of *trustworthy AI* was present in 4 focus groups. Some participants (n=5) had generally negative views of technology and AI, often citing that they simply do not trust AI-based technologies. A few participants (n=3) shared a broader dislike of technology.

*I think people put too much trust in technology.*

[P4, FG 1]

*I don’t love technology anyway though.*

[P17, FG 5]

A smaller subset of participants (n=2) raised concerns that aligned more closely with dystopian portrayals of AI.

*I already feel…mixed feelings I have about it. Societally, sometimes I get scared, that AI is going to be like what they show us in movies where, you know, it gets so close to actually being like a human.*

[P9, FG 2]

## Discussion

### Principal Results

#### Overview

Deception in mental health therapy is not a novel or hypothetical phenomenon, nor is its recognition limited to formal assessment tools. Our previous work has shown that while deception occurs infrequently in therapy, clinicians routinely encounter it in practice and already rely on informal strategies, such as noticing verbal inconsistencies, attending to behavioral cues, and engaging clients in direct conversation, to navigate moments of suspected nondisclosure [31]. Importantly, this literature emphasizes that clinicians are not positioned as interrogators but as relational actors who balance trust, autonomy, and care when deception arises. This study builds on this foundation by shifting focus from whether deception occurs or how clinicians detect it to examining how individuals who hold dual roles as clinicians and clients perceive the ethical, relational, and practical implications of introducing AI-enabled deception detection into an already complex therapeutic process.

An important tension emerging from this work concerns how deception is defined across technical and therapeutic contexts. Automated deception detection systems typically operationalize deception as intentional falsification of information. In contrast, participants in this study often described client “lying” as a form of self-protection or delayed readiness to disclose, rather than as malicious or deceptive intent. This framing is consistent with our previous findings, which showed that clinicians frequently understand nondisclosure in therapy as an adaptive response shaped by safety and trust [31]. Participants in the present study expressed concern that AI systems may fail to account for this nuance, potentially flagging clinically meaningful forms of guardedness as deception in ways that conflict with therapeutic norms. This tension sharpens the ethical challenge of deploying automated deception detection in mental health settings. Several key themes emerged, offering insights into how such technology could be implemented safely and ethically while also highlighting significant challenges to its integration. While themes emerged across participants’ dual roles as clinicians and clients, certain concerns were more salient when participants reflected on their client experiences (eg, perceived disruption), whereas others were more closely tied to professional authority and responsibility (eg, power dynamics).

Although the sample was not demographically diverse, its composition reflects key characteristics of the US mental health workforce. National workforce data indicate that psychologists in the United States are predominantly White and female. As such, the perspectives represented here align with those of a substantial portion of practicing clinicians, even as they do not capture the full range of identities present in the field. Combined with participants’ dual roles as clinicians and active therapy clients, this sample offers insight into how widely represented segments of the clinical community may experience the ethical and relational implications of AI-enabled deception detection, while also underscoring the importance of future work that centers more diverse clinician and client populations.

#### RQ1: What Ethical Frameworks and Safeguards Are Needed for the Responsible Integration of AI-Enabled Deception Detection in Therapeutic Settings?

Across focus groups, participants raised specific concerns about how AI-enabled deception detection might affect the therapeutic process. While some participants could envision potential benefits, especially in telehealth contexts, the dominant sentiment was caution. Many emphasized that any integration perceived as intrusive, judgmental, or disruptive would likely be incompatible with the values and goals of effective therapy.

As noted, participants appeared more open to the idea of AI-based deception detection within telehealth environments, potentially reflecting their general comfort with videoconferencing and online communication tools, which received the highest average rating across all technology categories (μ=4.5). Integrating the technology into platforms clinicians already use in virtual therapy could ease adoption, suggesting that embedding new tools within familiar systems may reduce friction. However, participants commented that any in-session alerts or technological distractions could interrupt clinician presence, undermine rapport, and shift the clinician’s focus away from the client. This aligns with broader concerns in the literature about how poorly timed or visible AI interventions can disrupt the therapeutic alliance and reduce the efficacy of treatment [32].

In addition to technical disruptions, participants noted the potential psychological harm of introducing automated deception detection. When digital systems are difficult to use or misaligned with client expectations, they can inadvertently provoke feelings of inadequacy or shame. As previous studies have shown, these technological barriers can become internalized, increasing self-stigma and discouraging help-seeking behaviors [33]. In our study, participants feared that adding deception detection technology could amplify this dynamic, particularly for clients with lower digital literacy or histories of trauma. Rather than supporting therapeutic progress, the technology might foster mistrust, inhibit disclosure, or lead clients to disengage altogether. These potential barriers were reflected in participant descriptions of the technology as a “third party” in the room (P14, FG 4), contrasting the metaphor by Haber et al [18] of AI as a benign, or even whimsical, “artificial third” in the therapeutic space. Participants in this study associated it more with surveillance, invoking imagery of a “watchful eye” or “Big Brother.” These concerns were consistent with participant ratings of comfort level with generative AI chatbots, which averaged 2.8 (SD 1.08), between “uncomfortable” and “neither comfortable nor uncomfortable,” indicating notable ambivalence. This discomfort with being watched or judged by a nonhuman system appeared to compound existing concerns about clinical power dynamics.

A central theme across all groups was the importance of client autonomy in the use of AI-enabled deception detection. Participants stressed that consent should be dynamic and revisited throughout treatment, allowing clients to opt in or out of the technology by topic, session, or altogether. This aligns with therapeutic models that view clients as experts of their own experiences and prioritize trust-based, gradual disclosure [34]. Within this framework, several participants emphasized that deception in therapy often functions as a protective act, an effort to maintain emotional safety rather than to mislead. Farber et al [7] similarly note that clients may deceive to shield themselves from judgment, rejection, or emotional pain. As one participant put it:

*I know that I have lied in therapy. It’s usually for some form of protection.*

[P1, FG 2]

From this perspective, the goal of therapy is not to “catch” deception but to foster a safe environment where clients feel ready to share their truth over time.

#### RQ2: What Technical and Procedural Safeguards Are Necessary to Preserve Client Confidentiality When Using AI in Therapeutic Contexts?

Confidentiality emerged as a central concern across all focus groups. Participants voiced deep unease about how data generated by AI-enabled deception detection, particularly data inferred from unconscious behavioral cues, would be handled, stored, and potentially accessed. Their concerns reflected not only a desire to uphold professional ethical standards, but also a broader fear that integrating this kind of AI system could introduce new risks to privacy, especially if protections were unclear or insufficient.

Participants emphasized that deception detection data should be treated with the same, if not greater, sensitivity as clinical notes or other health records. Several referenced the need for compliance with federal privacy laws, like HIPAA and the Cures Act, while others argued that the implicit nature of behavioral data might warrant even stronger safeguards. As 1 participant noted,

*This feels like even more precious, because it’s not even like a conscious report. It’s an unconscious piece of data.*

[P14, FG 4]

The possibility that such data could be misused in legal proceedings or accessed without consent, particularly through subpoenas or insurance claims, was a source of significant concern. We note that these concerns shared by participants reflect uncertainty regarding how these laws would apply to AI-generated behavioral signals rather than established legal precedent. Currently, the regulatory status of raw or derived AI-generated behavioral data remains an evolving policy question. Clarifying the governance of AI-generated behavioral data will require cross-disciplinary collaboration among clinicians, AI developers, and legal and policy experts, which is beyond the scope of this study but underscores the complexity of integrating AI into mental health care.

Participants also expressed uncertainty about whether and how these data would be stored. Some argued that deception-related outputs should not be retained at all, in part to protect clients from long-term consequences tied to misinterpretation or reuse of ambiguous data. Others believed limited storage might be acceptable if the information were encrypted and housed within secure, law-compliant platforms, and if clear boundaries were established about what would be retained and for how long. These views align with broader ethical discussions in digital mental health, which emphasize the importance of both data minimization and transparency in AI-driven systems [35].

From a procedural standpoint, participants highlighted the importance of informed and ongoing disclosure about data use, data access, and data sharing. Without this clarity, they warned clients might feel surveilled or exposed, particularly if they are already navigating complex legal, familial, or institutional contexts. These concerns were especially salient for clients in mandated therapy or high-risk populations, where AI-generated data might be used to influence custody arrangements, court proceedings, or clinical placements.

#### RQ3: What Design and Evaluation Strategies Can Prevent Bias and Promote Fairness in Therapeutic Applications of AI-Based Deception Detection?

Concerns about fairness and equity were among the most persistent themes across all focus groups. Participants repeatedly expressed apprehension that AI deception detection systems might reinforce or even amplify existing power imbalances within therapy, particularly for clients who already occupy vulnerable or marginalized positions. The fear was not just that the technology might misread behavior, but that it could do so in ways that disproportionately affected certain populations, undermining trust, reducing access, or skewing clinical decision-making.

One central concern was the potential for AI systems to misinterpret culturally specific behaviors as signs of deception. Participants questioned whether AI could reliably distinguish between genuine deceit and nonverbal expressions shaped by culture, neurodiversity, or emotional regulation strategies. As 1 participant noted,

*I think about people that have a lot of sarcasm...it may be perceived they’re being dishonest when they’re just being sarcastic.*

[P15, FG 4]

Others pointed out that expressions of discomfort that are commonly seen in therapy, could be easily conflated with deceptive behavior. This was especially relevant given treatment modalities like eye movement desensitization and reprocessing, which aim to treat traumatic memories and their effects [36,37], or other trauma processing that involves emotional dysregulation or avoidance [36] (P2, FG 1 and P12, FG 1).

Beyond these interpretability challenges, participants also expressed concern about the structure of the technology itself. Participants worried that the training data used to build deception detection models might not reflect the diversity of real-world therapy clients. This was especially salient given widespread knowledge of demographic skews in many machine learning datasets, which are often overrepresented by White, male, and Western participants [35,38-40]. As a result, AI systems may struggle to accurately interpret nonverbal and paralinguistic cues of clients from underrepresented backgrounds, leading to higher rates of false positives or interpretive errors for certain groups.

Participants were also mindful of how the introduction of the AI-based deception detection technology could shift the balance of power in the therapy room, a dynamic long recognized in psychotherapy literature as central to both ethical practice and client well-being [41,42]. If clinicians come to rely on AI judgments, particularly ones that are not easily explainable, it may diminish the client’s voice and agency. As 1 participant put it,

*If this thing is beeping at me...I’m being honest here, so that would also like introduce a layer of almost like a third entity in the room. It’s like I’m not mad at myself. I’m not mad at my therapist. I’m like mad at this AI thing.*

[P14, FG 4]

Others raised concerns about the appearance of conflict between clinician and AI feedback, which could erode the client’s confidence in both (P5, FG 6). Participants’ concerns about power imbalance can also be understood through the lens of epistemic injustice, where a person’s knowledge, testimony, or self-understanding is systematically discounted [43].

### Comparison With Previous Work

Unlike previous work on AI in psychotherapy, which has largely examined reflective or supportive systems (eg, chatbots [18], journaling aids [32,44], or expressive tools [45]), this study focuses on AI-enabled behavioral inference, specifically deception detection, within an active therapeutic relationship. This context introduces qualitatively different ethical risks; the system does not merely respond to client input, but interprets client behavior and intent, often without explicit client action. As a result, deception detection raises heightened concerns related to surveillance, misinterpretation of protective nondisclosure, power asymmetries, and downstream clinical or legal consequences. These features require safeguards that go beyond existing AI-in-therapy guidelines and motivate the need for context-specific refinements presented in this work.

The integration of AI into psychotherapy has been explored via conceptual frameworks, clinical case studies, and empirical surveys. Each investigation highlights unique challenges and opportunities relevant to the considerations and findings of this study. For example, Haber et al [18] conducted workshops and interviews with clinicians and clients to explore the role of generative AI chatbots in therapy. Their SAFE-AI framework emphasized transparency, participatory design, and patient safety, framing AI as an “artificial third” that can support and complicate the therapeutic relationship. Our participants agreed on the importance of transparency and autonomy but emphasized that, with deception detection, safeguards should be dynamic, allowing clients to opt in or out as needed. The interpretive nature of deception detection thus intensifies concerns Haber et al [18] identified in more reflective, supportive AI systems.

Building on this conceptual work, Haber et al [15] presented a proof-of-concept case study of customized OpenAI’s GPT-based tools (dialogic role-play-based externalization and visual externalization) that externalize clients’ internal experiences through dialogue and imagery. From this work emerged the SAFE-AI protocol, underscoring the importance of ethical oversight and clinical framing. Their study also treated AI as a tool for expression and exploration. By contrast, our participants described deception detection as a tool of judgment with the potential to surveil participants, inviting potential harm if misused.

A different methodological lens was taken by Prescott and Hanley [32], who surveyed both qualified and trainee therapists using a hypothetical “machine therapy” scenario paired with alliance measures and open-text responses. They found widespread skepticism, particularly regarding whether a machine could maintain the emotional “bond” central to the therapeutic alliance. Our participants echoed these anxieties, describing deception detection as a “third party in the room.” Yet, while the work by Prescott and Hanley [32] centered on AI therapy more broadly, our study revealed specific relational harms linked to surveillance metaphors and to the misinterpretation of protective client deception. In this way, our findings help to ground general concerns about rapport in the concrete dynamics of deception monitoring.

Reviews, such as the survey conducted by Poudel et al [35], have synthesized risks of AI in therapy at the systems level, highlighting issues of data privacy, bias, and the potential erosion of human connection. Their emphasis on encryption and governance offers essential baseline guidance. Our participants, however, voiced fears that go beyond abstract compliance, raising the specter of subpoenas, legal misuse, and the dangers of long-term retention of implicit behavioral data. To address these concerns, we recommend not only law-compliant storage but also time-limited retention and automatic deletion policies, aligning governance practices with the sensitivities of therapeutic trust.

Beyond AI-specific scholarship, substantial literature has examined the concept of deception itself in therapy. Dickens and Curtis [16] found that forensic therapists not only held inaccurate beliefs about behavioral cues to deception but also reported negative emotional responses when clients were found to be lying. Curtis [17] extended this by documenting therapists’ beliefs, attitudes, and even their own occasional use of deception, revealing that misperceptions and negative emotions are common. These findings underline the interpersonal weight deception that carries in clinical work. Our study adds to this picture by exploring how such tensions might be amplified or reframed when mediated by an AI system.

From the client’s perspective, deception is not rare. Curtis and Hart [8] surveyed therapy clients and found that most admitted to engaging in some form of deception, often via “white lies” or partial disclosures. Farber et al [7], drawing on two large client studies and decades of psychotherapy research, argued that deceptive behaviors often serve functional purposes, such as managing disclosure timing or preserving a sense of safety in the session. In our study, participants raised similar points, noting that these behaviors are not necessarily malicious but context-dependent. This observation highlights a potential risk for AI-enabled deception detection systems—without careful design, they may designate protective behaviors as deceptive. In turn, this could diminish client agency and disrupt a client’s ability to disclose at their own pace.

Finally, our own earlier work [5] explored clinician perspectives on deception detection, identifying broad concerns about accuracy, privacy, and disruption. The previous study, however, reflected only one side of the therapeutic dyad. By incorporating participants who are both clinicians and clients, this study surfaces new insights about power imbalances, stigma, and cultural sensitivity, and translates them into concrete recommendations—dynamic consent, nonpunitive interpretation, short retention windows, and alliance-protective integration.

While previous work on AI in therapeutic contexts, particularly chatbot- and reflection-based systems, emphasizes transparency, empathy, and participatory design, our findings show that these principles are insufficient when applied to AI systems that draw conclusions about clients’ honesty or intent without their explicit disclosure. Deception detection introduces qualitatively different ethical risks. It disrupts disclosure timing, produces involuntary behavioral inferences, and alters power dynamics within the therapeutic relationship. As a result, our study extends existing guidelines by specifying new requirements for dynamic, revocable consent; nonintrusive, postsession system integration; data minimization and nonretention of inferred behavioral signals; context-sensitive fairness evaluation; and explicit safeguards against adjudicative authority. These recommendations are not incremental refinements of previous frameworks but emerge directly from the unique interpretive and judgment-bearing nature of deception detection in therapy.

Taken together, these studies show a recurring emphasis on autonomy, transparency, and alliance protection as prerequisites for ethical AI in therapy. Our contribution complements this work by grounding those principles in the specific and sensitive context of deception detection. In doing so, we translate them into practice-focused and stakeholder-informed safeguards aimed at promoting AI that supports, rather than undermines, therapeutic care.

### Implications for Design and Practice

#### Overview

Across the major themes (ie, integration, data confidentiality, and AI integrity), participants emphasized that any AI system introduced into therapy must integrate seamlessly into clinical workflows, preserve client autonomy, protect confidentiality, and proactively address cultural representation and power dynamics. Although these concerns emerged in the specific context of deception detection, they reflect broader expectations for ethical AI integration in mental health care. Participants’ emphasis on autonomy and disclosure timing highlights the importance of human-centered design approaches that meaningfully involve both clinicians and clients throughout system development. Such collaborative design processes may increase tool alignment with therapeutic goals while reducing chances of technological disruption.

Furthermore, participant concerns related to consent, transparency, and data governance underscore the need for ethics frameworks that explicitly address these dimensions when evaluating AI tools in clinical contexts. Participants’ fears of trust erosion, false positives, and power imbalances suggest that evaluation must extend beyond technical performance to consider relational impact, therapeutic outcomes, and mechanisms for clinician and client feedback. Clinicians’ ability to appropriately interpret and explain AI outputs also emerged as critical; without adequate training, institutional guidance, and supervisory support, there is a risk of misinterpretation or overreliance that might undermine both professional judgment and client trust. Although this study did not examine alignment with standardized psychological assessments that include deception-related or validity indicators, such measures may serve as useful reference points during system development and evaluation, without implying equivalence to or substitution for formal clinical testing. Drawing from these cross-cutting principles, we recommend the following safeguards and implementation strategies to support responsible integration.

#### RQ1: Client Autonomy and Therapeutic Integrity

Findings related to RQ1 underscore that deception detection introduces relational and disclosure-timing risks that require careful ethical integration. In response, we recommend adopting flexible, dynamic, and revocable consent models that allow clients to opt in or out of the technology over time, including by session or topic. Providing clear, comprehensible explanations of system functionality, data collection, output generation, and retention practices preserves client agency and reduces perceptions of surveillance. Treating consent as an ongoing process directly addresses participant concerns that static disclosures may undermine trust.

Participants also indicated that prioritizing postsession review rather than real-time alerts may directly address concerns that in-session notifications may disrupt clinician presence and weaken therapeutic rapport. In contrast, postsession summaries may preserve therapeutic flow while allowing space for clinical discretion. For example, after a session, a clinician might privately review a high-level summary indicating portions of the session associated with elevated uncertainty, without labeling specific statements as lies or identifying precise time stamps. Such information may inform reflection on pacing, trust-building, or gradual exploration while remaining grounded in the therapeutic context.

Framing deception detection explicitly as a supportive and optional tool reinforces its suggested function as a clinician-guided aid rather than a diagnostic authority or surveillance mechanism. This positioning aligns with ethical principles in digital mental health applications, including transparency, harm reduction, and client empowerment [15,20]. For AI developers, these findings suggest that client-facing interfaces prioritize granular, moment-to-moment control rather than binary opt-in consent. For instance, a client control panel may allow clients to temporarily pause inference during sensitive topics and review plain-language explanations of system outputs at their discretion. Such controls reinforce that the system remains optional and subordinate to therapeutic goals.

#### RQ2: Confidentiality, Data Governance, and Institutional Safeguards

Concerns about data protection in response to RQ2 centered on emphasizing the heightened sensitivity of AI-generated behavioral inference data and the potential for downstream misuse. In response, we recommend implementing multilayered safeguards for data protection and access control. Encrypting behavioral inference data and storing it only on platforms meeting applicable legal and industry standards (eg, HIPAA and General Data Protection Regulation) aligns with participant concerns regarding privacy and unauthorized access. Automatic deletion after a short, predefined period (eg, 30 d), unless clinically justified, further mitigates long-term risk and reduces perceptions of continuous monitoring.

As for data access, limiting access by default to the treating clinician—and, where appropriate, a designated supervisory or ethics role—maintains consistency with established norms governing psychotherapy records [33,34]. Restricting access by commercial vendors, administrators, insurers, or legal entities without explicit client consent reinforces the principle of minimal necessary access. Clear specifications that define the scope and limits of data use within consent materials address fears of downstream misuse. For clinic directors and administrators, consent forms may state that AI-generated data are used solely within therapeutic care, are not used to train external AI systems, and do not serve legal or disciplinary functions. Participants emphasized that such clarity reduces the likelihood that the technology will be perceived as a surveillance or enforcement tool.

#### RQ3: Fairness, Algorithmic Bias, and Procedural Deployment

Issues expressed regarding fairness extended beyond model bias to include concerns about inequitable deployment and power asymmetries. To mitigate structural bias, we recommend prioritizing representative model development and context-sensitive validation. Training models on datasets reflecting diverse racial, ethnic, cultural, gender, and neurodivergent identities aligns with current algorithmic fairness standards [21,32]. However, participants emphasized that extending evaluation beyond aggregate accuracy might help to address risks that clinically normative behaviors may be misclassified as deception. In addition to reporting disaggregated false positive and false negative rates across demographic groups, researchers should incorporate therapeutic-context metrics, such as error rates during trauma processing or high distress, performance differences across therapeutic modalities (eg, assessment vs trauma-focused sessions), and the longitudinal stability of outputs within individuals. These additional metrics strengthen empirical accountability by identifying whether systems disproportionately flag normative clinical behaviors as deceptive, even when demographic fairness criteria appear satisfied.

Participants also raised concerns regarding procedural fairness in deployment. Clear, equitable use policies are essential. Selective deployment—such as limiting use to certain clients or high-risk contexts—may introduce or exacerbate disparities. Although previous work suggests that honesty is generally the default in interpersonal interactions, including psychotherapy [46,47], deception has been shown to cluster around stigmatizing or high-stakes topics, such as suicidal ideation and substance use [7,8]. While some participants viewed context-sensitive use as preferable to broad deployment, others questioned the fairness and feasibility of “picking and choosing” when and with whom such tools should be applied. Participants expressed concern regarding decision-making authority, justification criteria, and the potential for discretionary deployment to introduce new forms of bias, stigma, or power imbalance within the therapeutic relationship.

To address these risks, we recommend that institutions establish transparent guidelines defining when and how the technology may be used. Developing such policies in consultation with clinicians, clients, and ethicists reinforces procedural equity and institutional accountability.

### Limitations

This study has several limitations that should be considered when interpreting its findings. First, the sample was small (n=18) and demographically skewed, with most participants identifying as White women within a similar age range, and a large student representation. We attribute this skew to the inherent female-domination in the profession [28] and to our recruitment strategy, which relied heavily on publicly available contact information from student listservs, counseling centers, and faculty at universities. As a result, the perspectives shared may not fully reflect the diversity of views held by therapists and therapy clients across different racial, gender, or cultural backgrounds. Second, although focus groups can offer rich and dynamic discussions, participants were not grouped based on specific characteristics, such as work setting, therapeutic orientation, or demographic background. This was due to scheduling constraints and the need for flexibility in participant availability. While this open grouping allowed for diverse exchanges, it may have limited opportunities to explore context-specific or identity-based patterns in depth. Third, group sizes varied from 2 to 5 participants. While smaller groups can allow for deeper individual reflection, they may lack the energy and interplay typical of larger focus groups, which can affect the types of insights that emerge. Additionally, not all groups addressed the same topics equally. Although common threads were identified across sessions, certain themes surfaced organically in some discussions but not others; this scenario is typical of focus group research. Finally, while wanting to present oneself favorably or match the opinions of the group is a potential concern in focus group settings, the individuals in this study appeared comfortable offering candid, critical, and nuanced perspectives, which helps to mitigate this concern.

### Conclusions

This study examined the perspectives of individuals who occupy the dual roles of mental health clinician and current therapy client to explore the ethical, relational, and practical implications of integrating AI-enabled deception detection into therapeutic contexts. Through 6 virtual focus group sessions, participants identified potential benefits alongside significant concerns related to disruption of the therapeutic process, erosion of client autonomy, confidentiality risks, and the potential for bias or inequitable application. These insights highlight the importance of dynamic, client-driven consent models, culturally sensitive design, and careful consideration of how such tools are introduced and used in practice.

While AI-enabled detection technology has been proposed to possibly augment clinical insight [5], key findings of this study suggest that its integration into therapy must be guided by a commitment to safe, ethical practice. Participants expressed concern that without thoughtful safeguards, these tools risk harming the very people they are intended to support.

Although this study focused on deception detection, the concerns voiced by participants around therapeutic disruption, client autonomy, data security, and fairness likely apply broadly to many forms of AI technologies used in mental health care. As AI tools continue to enter therapeutic spaces, these findings underscore the importance of developing integration strategies that are aligned with clinical values and responsive to both clinician and client perspectives.

Ultimately, addressing these challenges will be essential to ensuring that AI-enabled technologies support, rather than undermine, the core values of mental health care—trust, safety, autonomy, and connection.

## Supplementary material

10.2196/86633 Multimedia Appendix 1 Participant demographics by participant ID and focus group number.

10.2196/86633 Multimedia Appendix 2 Copy of focus group guide used in all 6 focus groups.

10.2196/86633 Multimedia Appendix 3 Codebook developed via qualitative analysis and including coding structure and definitions.

10.2196/86633 Multimedia Appendix 4 Code frequencies by participant, with their demographic status as students or nonstudents.

## Abbreviations

AI: artificial intelligence
EMR: electronic medical record
HIPAA: Health Insurance Portability and Accountability Act
SAFE-AI: Screening, Alignment, Facilitation, and Evaluation of AI-Enhanced Interventions
