# Supplementary material for: Ethics and Fairness Considerations in AI-Based Deception Detection Technologies for Mental Health Applications: Focus Group Study
Source: JMIR AI. 2026 May 25;5:e86633. doi: 10.2196/86633 (PMC13200768; doi:10.2196/86633)
Supplement: Multimedia Appendix 2 [file ai-v5-e86633-s002.docx]

**Study Introduction:**

We have been investigating the impacts of client deception in mental health settings. In particular, we interviewed practicing mental health clinicians about their experiences with client deception and their perceptions of leveraging AI to detect deception during therapeutic sessions. Largely, the clinicians we interviewed had concerns about the impact of such a tool on a client’s autonomy, the potential harm to the therapeutic alliance, and other ethical implications.

The purpose of this focus group is to understand how to address the ethical concerns and considerations that we uncovered in our previous study. We also hope to gain insights that will inform how to safely and ethically integrate AI-enabled deception detection technologies in therapeutic sessions.

You all have a unique perspective since you are actively treating clients as a clinician and identify as someone actively seeking clinical treatment/therapy for a mental health challenge(s). Throughout this conversation we are eager to hear your thoughts from your perspective both as a clinician and as a client. We invite you to elaborate on the topics we discuss from either perspective or from both.

Do you have any questions so far?

Some guiding principles for the session:

We want to remind you that there are no wrong answers. We are really here to learn from you. Feel free to discuss, agree, contrast, and share personal experiences to help illustrate a point or idea that you have. Please do not be shy about sharing what may be perceived as negative comments they are just as valuable as positive comments.

We do ask that you are mindful of your response time throughout the session – it’s important for us to hear from you all so I'll be kinda tracking our time as we move from question to question.

Currently, we are audio-visually recording via teams. We will get a transcript from this recording that we will retain and remove identifying information from that transcript. Regarding the videos, we will re-watch, take notes extract the crux of what you will share today, and then discard the videos. So please feel free to communicate freely – no identifying information will be linked to what you say here today.

If you haven’t already, please silence your phones. We just want to ensure that we minimize possible interruptions in the recording that might cause us to miss anything while someone is talking.

*Opening:*

(Round robin)

Talk about your professional role, what a typical day looks like for you, and what do you hope to get out of your treatment as a client?

*Intro:*

How do you define ethics?

*Transition:*

How do you consider ethics in your daily life and within your role?

AI Technology Description:

We are considering the use of AI-enabled deception detection technology that can be used during sessions to provide the clinician with real time notifications of when a client is likely being dishonest when discussing important treatment-related information. Some examples of possible similar implementations of this tool include a Google Home/Amazon Echo that leverages characteristics of speech, or perhaps a smartwatch that analyzes physiological information and similar speech information, or something that runs in the background on a virtual session that analyzes body language and speech characteristics. These are just examples to help you conceptualize the possible implementations for the technology to help you better answer the following questions should focus on ethical considerations.

*Key:*

How might a tool like this be integrated into your current workflow as a clinician while respecting client confidentiality?

FU: For those that have mentioned federal regulations, what aspects should these regulations address to protect client confidentiality?

If this tool were used during therapeutic sessions, what thoughts come to mind regarding the preservation of client autonomy?

FU: What steps can be taken to mitigate the deterioration of client autonomy related to the aspects you’ve mentioned?

What should data access to the AI-tool's results look like?

What should be done to ensure the AI-tool is fair?

*We’ve been investigating this from a computational perspective, we were wondering if you think*  *you would expect certain impacts from demographic groups?*

*Ending:*

Out of everything we discussed today, what is the most important to you and can you think of any ethical points that I have not asked you about? (Round robin)

Feedback from participants

Comments from the moderator
